# Open Screw Placement in a 1.5 mm LCP Over a Fracture Gap Decreases Fatigue Life

**DOI:** 10.3389/fvets.2018.00089

**Published:** 2018-05-23

**Authors:** Sarah G. J. Alwen, Amy S. Kapatkin, Tanya C. Garcia, Joshua Milgram, Susan M. Stover

**Affiliations:** ^1^JD Wheat Veterinary Orthopedic Laboratory, School of Veterinary Medicine, University of California, Davis, Davis, CA, United States; ^2^ACCESS Specialty Animal Hospital, Culver City, CA, United States; ^3^Department of Surgical and Radiological Sciences, School of Veterinary Medicine, University of California, Davis, Davis, CA, United States; ^4^Department of Anatomy, Physiology, and Cell Biology, School of Veterinary Medicine, University of California, Davis, Davis, CA, United States; ^5^Koret School of Veterinary Medicine, The Robert H. Smith Faculty of Agriculture, Food and Environment, The Hebrew University of Jerusalem, Jerusalem, Israel

**Keywords:** condylar locking plate, radial fracture, toy-breed, biomechanical, ex-vivo, gap model

## Abstract

**Objective:**

To investigate the influence of plate and screw hole position on the stability of simulated radial fractures stabilized with a 1.5 mm condylar locking compression plate (LCP).

**Study Design:**

*In vitro* mechanical testing of paired cadaveric limbs.

**Sample Population:**

Paired radii (*n* = 7) stabilized with a 1.5 mm condylar LCP with an open screw hole positioned either proximal to (PG), or over (OG), a simulated small fracture gap.

**Methods:**

Constructs were cycled in axial compression at a simulated trot load until failure or a maximum of 200,000 cycles. Specimens that sustained 200,000 cycles without failure were then loaded in axial compression in a single cycle to failure. Construct cyclic axial stiffness and gap strain, fatigue life, and residual strength were evaluated and compared between constructs using analysis of variance.

**Results:**

Of pairs that had a failure during cyclic loading, OG constructs survived fewer cycles (54,700 ± 60,600) than PG (116,800 ± 49,300). OG constructs had significantly lower initial stiffness throughout cyclic loading and higher gap strain range within the first 1,000 cycles than PG constructs. Residual strength variables were not significantly different between constructs, however yield loads occurred at loads only marginally higher than approximated trot loads. Fatigue life decreased with increasing body weight.

**Conclusion:**

Fracture fixation stability is compromised by an open screw hole directly over a fracture gap compared to the open screw hole being buttressed by bone in the model studied. The 1.5 mm condylar LCP may be insufficient stabilization in dogs with appropriate radial geometry but high body weights.

## Introduction

Bone plate fixation is commonly used to stabilize distal radial fractures in toy breed dogs (1). However, reported complications after fixation include higher frequencies of delayed union and nonunion when compared with larger dogs treated identically for similar fractures (2, 3). Insufficient fracture fixation stability in toy breed dogs could contribute to the higher frequency of complications.

Implant selection for internal fracture stabilization is limited by radial and fracture geometry in toy breed dogs. Radial medioateral diameter in dogs less than 7 kg ranges from 6 to 6.4 mm (4). It has been shown in cadaveric sheep femora that ultimate failure torque decreased linearly with increasing size of unfilled unicortical defects, with a 1/2 reduction in failure torque when defect diameter was 40% of bone diameter (5). While filling the hole with a screw is expected to minimize this reduction (6), only 1.5 mm screws and plates meet the recommended AO principle that drill holes are less than 40% of bone diameter to minimize risk of iatrogenic bone fracture (7). Secondly, the typical short length of the distal fragment in toy-breed distal radial fractures can require a T-plate or condylar plate configuration to ensure that at least 2 plate screws engage the distal fragment. Thirdly, locking screw plate fixation of cadaveric porcine metacarpal fractures achieved greater bending fixation stiffness and load to failure than non-locking screw plate fixation (8). Therefore, a locking plate design may achieve greater fracture repair stability in toy breed dogs than non-locking plate systems in radii which sustain bending during axial loading.

The 1.5 mm condylar LCP system** ®** satisfies the geometry, dimension, and loading considerations for cranial plate fixation of distal radial fractures, and is used clinically, in toy-breed dogs. The plate is designed to allow interfragmentary compression at hole #6, achieving compression at the fracture if used and locked fixation in the small distal fragment and the shaft of the plate. In clinical situations, fracture obliquity can preclude filling the compression hole with a screw due to the common position of the fracture line directly below the cortex screw hole in order to properly accommodate the T portion on the small distal fragment. Therefore, despite careful positioning of the 1.5 mm condylar LCP on the radius to accommodate the T portion of the plate on the distal fragment, the cortex screw hole of the 1.5 mm condylar LCP often coincides fully or partially with the fracture line and is left unfilled.

Plate failure is assumed to be more likely at empty screw holes because high stresses occur at these regions of low cross-sectional plate area (9, 10). Although clinical recommendations are never to have an empty screw hole directly over the fracture line, it is unknown whether an empty screw hole partially or fully buttressed by the bone would have some protective function against plate failure. The objective of this study is to investigate the influence of plate and screw hole position on the biomechanical properties in a gap model of simulated toy breed radial fractures stabilized with the 1.5 mm condylar LCP. It was hypothesized that the fatigue properties and residual bending strength of distal radial fractures stabilized with a 1.5 mm condylar LCP in toy-breed dogs will be compromised if an open screw hole is placed directly over the bone fracture.

## Materials and Methods

### Study Design

The effects of the position of a plate, specifically of an open screw hole, relative to the fracture gap (proximal, over) on biomechanical properties of canine radii stabilized with a 1.5 mm condylar LCP were evaluated using plate stabilization of a simulated distal radial fracture with a gap in 7 pairs of cadaveric radii, with the 2 different treatments in contralateral radii. Cadavers were from two institutions and all died or were euthanized for reasons unrelated to the study. All cadavers in this study were approved for research. Construct stability was evaluated by simulating axial cyclic loading and residual strength in a mechanical testing system and by quantifying construct stiffness, fatigue life, and residual strength, and gap strain.

### Constructs

**Figure 1:**
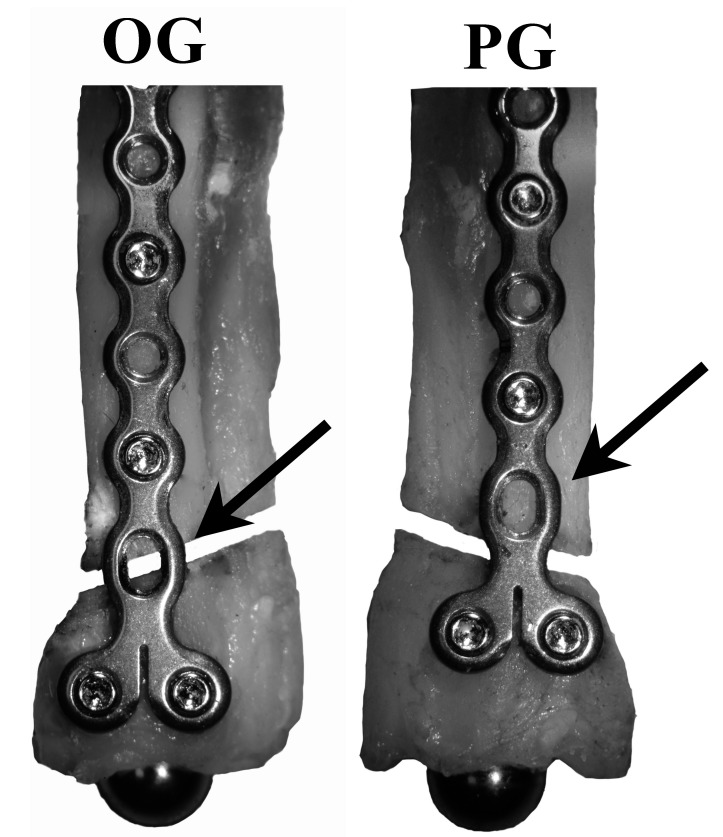
Treatment groups (OG, Over the Gap; PG, Proximal to the Gap) with cranial and medial views of plate and screw positions. Black arrow: the unfilled #6 cortex screw hole is centered over (OG), or proximal (PG) to the simulated fracture. All groups have 1.5 mm diameter locking screws placed in screw positions 1, 3, 5, 7, and 8.

Two constructs, one each on contralateral radii from each cadaver, were evaluated. Both constructs used a 1.5 mm, 8 hole condylar LCP (DePuy Synthes Vet, 1301 Goshen Parkway, West Chester, PA 19380) and 5, 1.5 mm locking screws (DePuy Synthes Vet, 1301 Goshen Parkway, West Chester, PA 19380) placed on the cranial aspect of the radius in accordance with manufacturer guidelines to stabilize a simulated complete oblique distal fracture with a gap^1^. Plate holes 1, 3, 5, and those in the T portion were filled while plate holes 2, 4, and 6 were left unfilled. The 2 constructs varied by plate position that caused the position of open screw hole 6 to change relative to the simulated fracture gap (Figure 1).

### Specimen Preparation

Bicortical holes were drilled in plate holes 1, 3, 5, 7, and 8 using a 1.1 mm diameter drill bit through the appropriate 1.5 mm locking drill guide securely threaded into the 1.5 mm condylar LCP. Self-tapping 1.5 mm locking screws were inserted bicortically in drilled holes. All screws were placed and hand-tightened by an experienced board-certified surgeon (ASK) in accordance with the manufacturers guidelines for the 1.5 LCP technique^1^. After fixation, a standardized oblique simulated fracture coursing proximolateral to distomedial was created using a dremel tool and hand saw being careful not to damage the plate. The resulting osteotomy gap was 2 mm thick (thickness of the dremel tool blade) and was cut obliquely using standardized landmarks (16.5 and 18.1% of bone length, measured by a electronic caliper, from the distal most extent of the bone for the medial and lateral sides, respectively) in a plane perpendicular to the frontal plane through the bone. Bone length was measured from proximal to distal aspects of the radius. Bone medio-lateral and cranio-caudal width were measured at 25% bone length from the distal-most aspect of the radius (near the gap) with an electronic caliper.

To allow bending of the specimen in the sagittal plane during axial loading due to the curved geometry of the radius, a hemispherical metal ball (1/4” ball, 250-TBR-T, Bal-Tec, Los Angeles, CA) on a threaded post (#4–40 set screw, McMaster-Carr, Santa Fe Springs, CA) was attached to the distal radial segment. The ball interfaced with a custom concave ¼” hemispherical cup attached to the mechanical testing system loading plate. The proximal end of the radius was rigidly secured in a pot for attachment to the mechanical testing system using transfixation K-wires and polymethylmethacrylate (PMMA) (Coe Tray Plastic, GC America). Alignment of the radius within the pots to ensure axial loading along the longitudinal axis of the radius was achieved using 2 orthogonal lasers. The PMMA was allowed to set for 30 min before testing.

**Figure 2:**
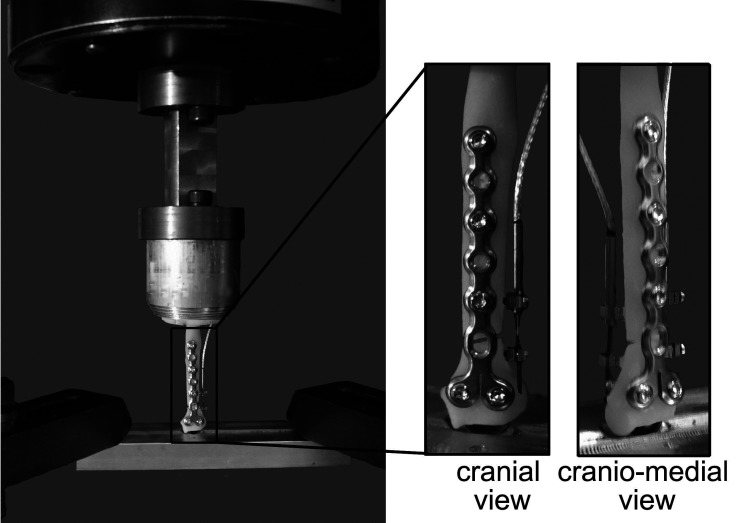
Materials testing system illustrating a loaded toy breed radius model with a simulated 2 mm fracture gap and cranially applied 1.5 mm condylar LCP. One linear displacement transducer was positioned laterally and one caudally. The distal end of the radius is fixed with a stainless steel hemispherical ball to allow for rotation and bending in all directions. The proximal end is held rigid.

Two linear displacement transducers (DVRTs; Model M-DVRT-3, LORD MicroStrain, Williston VT) spanned the caudal and lateral aspects of the simulated fracture gap to measure gap displacement in the sagittal and cranial planes during specimen loading (Figure 2).

### Mechanical Testing

Constructs were cycled in axial compression in a materials testing system (Model 809, MTS Corporation) from 5–108% of cadaver body weight to simulate trot load (11, 12) under load control at 1.5 Hz until failure or a maximum of 200,000 sinusoidal cycles (approximate number of steps a small dog takes within 3–4 months) (13). Constructs were completely unloaded at 1,000 cycles, and after failure or 200,000 cycles if failure did not occur, to allow determination of residual displacements in unfailed specimens. Failure was defined as a construct displacement of 1.5 mm. Specimens that did not fail within 200,000 cycles were loaded in a single cycle in axial compression under load control at 20 N/s until failure. Axial load, construct (actuator) displacement, and for cyclic tests gap displacement, data were collected at 128 Hz during tests. Test failure was defined on the load versus actuator displacement curve as the point just prior to inability to sustain load.

### Data Reduction

Fatigue life was calculated as the number of cycles attained until the construct failed. Failure mode was categorized as plate breakage, screw-bone interface failure (screw loosening), screw-plate interface failure, or no apparent failure.

Gap strains (lateral, caudal) were calculated from respective gap displacements (δL) and original gap length (L); as strain (ε)= δL/L with L = 2 mm. Gap strains were compared between constructs for the first 1,000 cycles, i.e., before any construct failed during cyclic testing. Residual gap displacements after 1,000 cycles were calculated as the differences in gap displacements in unloaded constructs at the 1st and 1,000th cycles.

**Figure 3:**
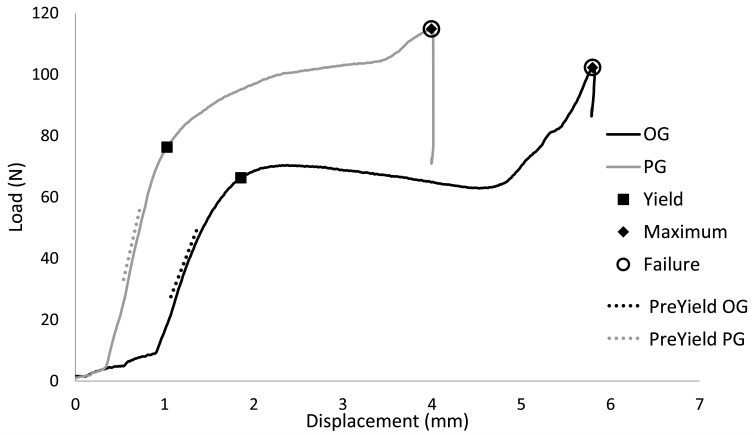
Representative load versus displacement curves indicating yield, maximum, and failure points during residual strength, single axial load to failure tests.

Residual strength mechanical testing variables were derived from load versus displacement curves (Figure 3). Variables included pre-yield stiffness, and loads, displacements, and energies at yield, maximum, and failure points. Stiffness was calculated by least squares regression of the middle 1/3 of the data before yield. The yield point was determined as the point where data first deviated from the pre-yield stiffness linear displacement intercept by 0.1% of the displacement intercept with custom software (MATLAB, Mathworks Natick MA). The point of maximum strength is the highest load before failure. The energies to yield, maximum and failure were calculated as the areas under the curve to the respective points on the curve.

### Statistical Analysis

The effects of plate position on cyclic fatigue life variables at specific cycles and on residual strength variables were assessed using a mixed model analysis of variance (α = 0.05). Repeated measures were accounted for within dog cadaver and the cadaver was treated as a random variable. Normality of the residuals from each ANOVA was checked with the Shapiro Wilks statistic. Data are reported as least square mean ± SE error. The relationships between gap strain range and fatigue life, and between dog body weight and fatigue life, were evaluated graphically and using Pearson’s correlation tests.

## Results

Bones from female spayed cadavers with a median age of 10 years (range, 2–13 years), median weight of 3.5 kg (range, 2.8–7.2 kg) and median radial length of 73.4 mm (range, 58.7–114.0 mm) were studied. There was no linear correlation between radius length and body weight (*p* = 0.13).

### Fatigue Life

**Figure 4:**
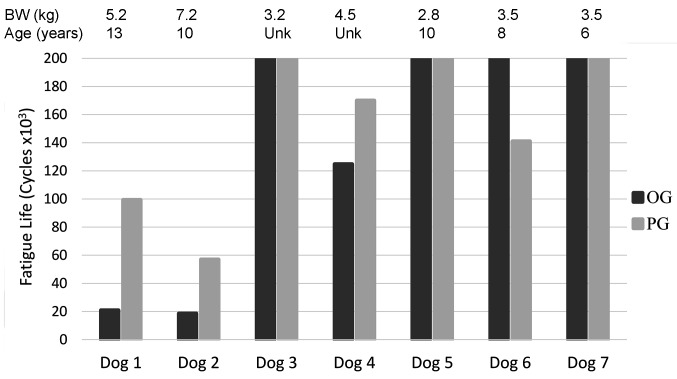
Fatigue life of paired OG and PG constructs through 200,000 cycles.

Of the 7 pairs of cadaveric radii tested cyclically, 3 pairs (OG and PG constructs) completed 200,000 cycles without failure (cadavers <3.5 kg), 3 pairs failed in <200,000 cycles (cadavers >3.5 kg), and in 1 pair only the PG construct failed in <200,000 cycles (cadaver = 4.2 kg) (Figure 4). Of paired constructs that had at least one failure before 200,000 cycles, 3 of 4 OG constructs failed in fewer cycles (54,700 ± 60,600, mean ± SD) than their contralateral PG (116,800 ± 49,300) constructs.

The most common mode of failure was plate breakage at the level of the fracture gap followed by screw-bone interface failure in the distal bone fragment. One construct exhibited no visual evidence for cause of failure (Table 1).

**Table 1:** Mode of failure during fatigue tests by construct type for those constructs that failed in fewer than 200,000 cycles.

**Mode of Failure**	**OG Constructs**	**PG Constructs**	Total*
Plate breakage	2	3	**5**
Screw-bone interface	1	1	**2**
Screw-plate interface	0	0	**0**
None apparent	1	0	**1**

* The total amounts to more than 7 bones (the number of bones that failed during cyclic testing) as a result of dual modalities of failure in one bone tested.

### Residual Gap Displacement at 1,000 Cycles

OG constructs had a greater residual gap displacement than contralateral PG constructs in 5 out of 7 cadavers. The difference in mean residual gap displacement between constructs at 1,000 cycles (OG: 0.31 ± 0.30 mm vs PG: 0.16 ± 0.33 mm) was not significant (*p* = 0.075).

### Construct Stiffness

**Figure 5:**
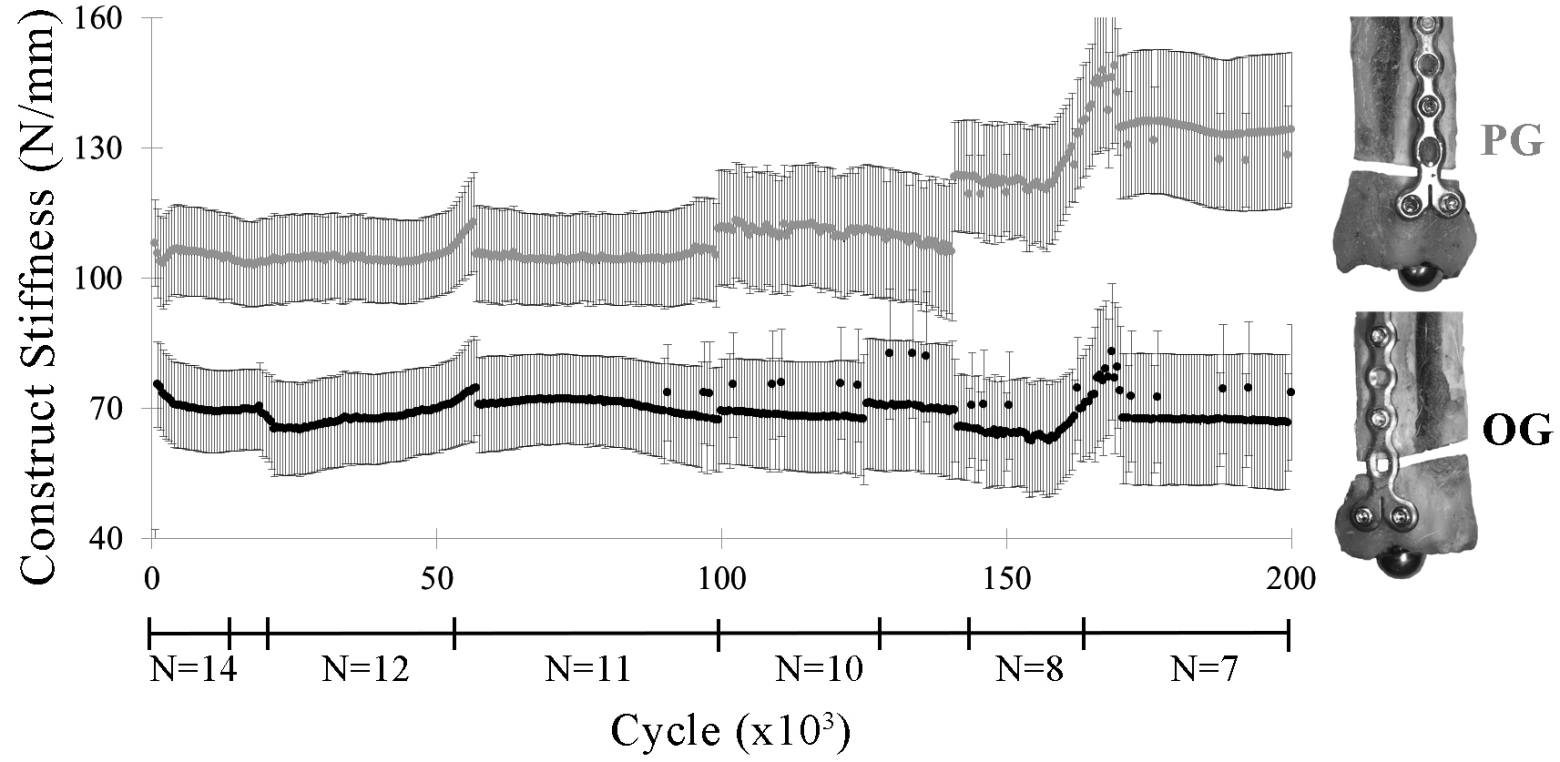
Construct stiffness (lsmean ± SE) by construct (PG, OG) through 200,000 cycles. Irregularities in the graph correspond to construct failures before 200,000 cycles, as indicated by the construct sample sizes (N) below the *x*-axis.

Construct stiffness was significantly greater in PG constructs versus OG constructs for most cycles through 200,000 cycles (*p* ≤ 0.01) (Figure 5). The increase in stiffness during cyclic loading after approximately 150,000 cycles coincided with the failure of specimens of lower stiffness.

### Gap Strains

**Figure 6:**
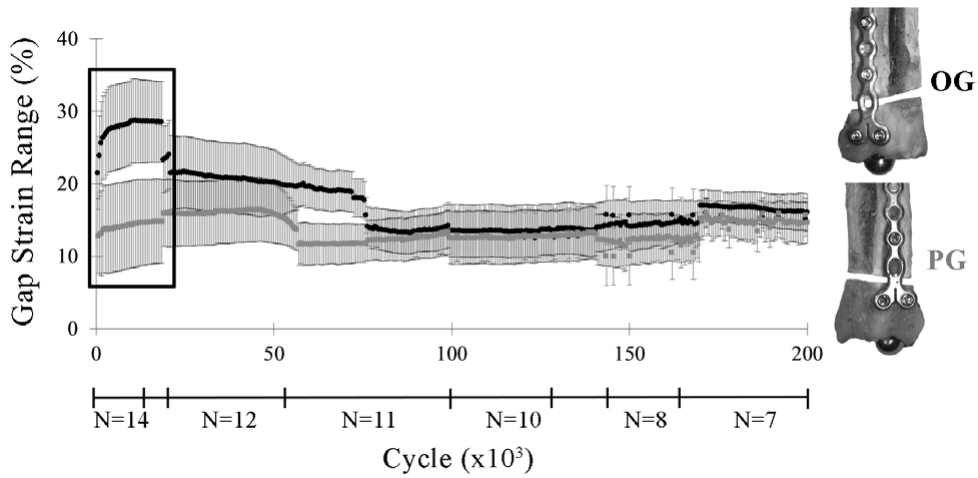
Caudal gap strain range within each cycle (lsmean ± SE) by construct (OG, PG) through 200,000 cycles. The largest difference in gap strain range occurred during the first 20,000 cycles (box). As the less stable constructs drop out due to early failure, differences between constructs in gap strain range were no longer apparent.

OG constructs had significantly greater ranges in caudal gap strain within cycles during the first 1,000 cycles than PG constructs (*p* ≤ 0.02; Figure 6). There were no significant differences in gap strains laterally or between constructs at either high or low levels of the cyclic loads within each cycle after the first 1,000 cycles (high load strain: OG 25.5–8.5%, PG 15.8–8.7%, *p* = 0.11; low load strain: OG 2.9–3.6%, PG 2.9–3.7%, *p* = 0.68).

**Figure 7:**
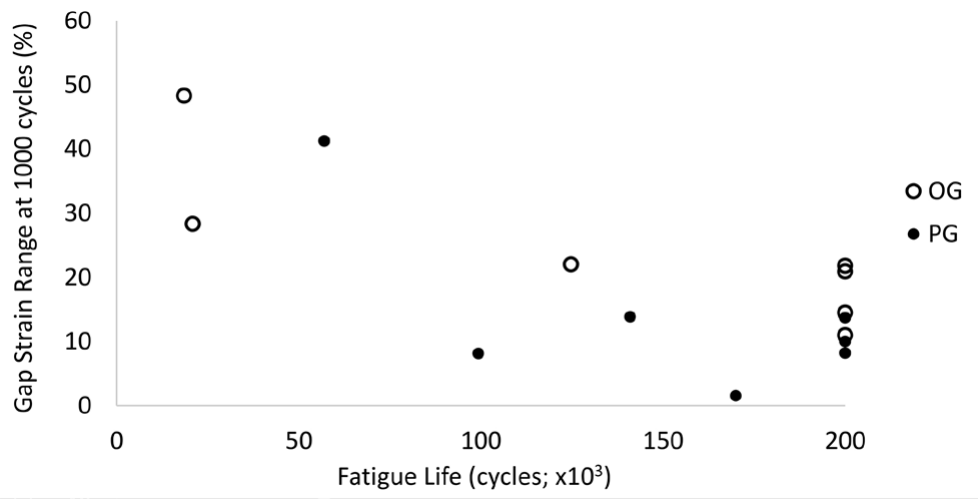
Caudal gap strain range at 1,000 cycles vs fatigue life for OG and PG specimens.

There was a statistically significant inverse relationship between fatigue life and caudal gap strain range at the end of the first 1,000 cycles (R = −0.73; *P* = 0.003; Figure 7).

### Residual Strength

There were no statistically significant differences in stiffness, or yield, maximum, or failure displacements, loads, or energies between constructs (Table 2). All constructs failed during residual strength testing due to plate bending.

**Table 2:** Residual strength test variables lsmean (95% Confidence Limits) for OG and PG constructs.

Variable	OG	PG	*p*-value
Sample size	4	3	
Pre-yield stiffness (N/mm)	52 (14,90)	109 (66,153)	0.052
Yield			
Displacement (mm)	2.0 (1.1,2.8)	0.9 (−0.1,1.9)	0.089
Force (N)	80 (57,105)	73 (47,100)	0.573
Energy (kN*mm)	90 (28,153)	41 (−31,113)	0.267
Maximum			
Displacement (mm)	7.5 (4.8,10.2)	5.9 (3.0,8.8)	0.249
Force (N)	405 (−34,844)	352 (−82,786)	0.329
Energy (kN*mm)	1,330 (−381,3024)	967 (−805,2736)	0.367
Failure			
Displacement (mm)	8.1 (5.0,11.1)	6.5 (3.3,9.8)	0.328
Force (N)	324 (−19,666)	329 (−11,669)	0.835
Energy (kN*mm)	1,584 (−342,3510)	1,204 (−802,3209)	0.438

*Table S1 is residual strength test values for individual OG and PG constructs.

### Body Weight

**Figure 8:**
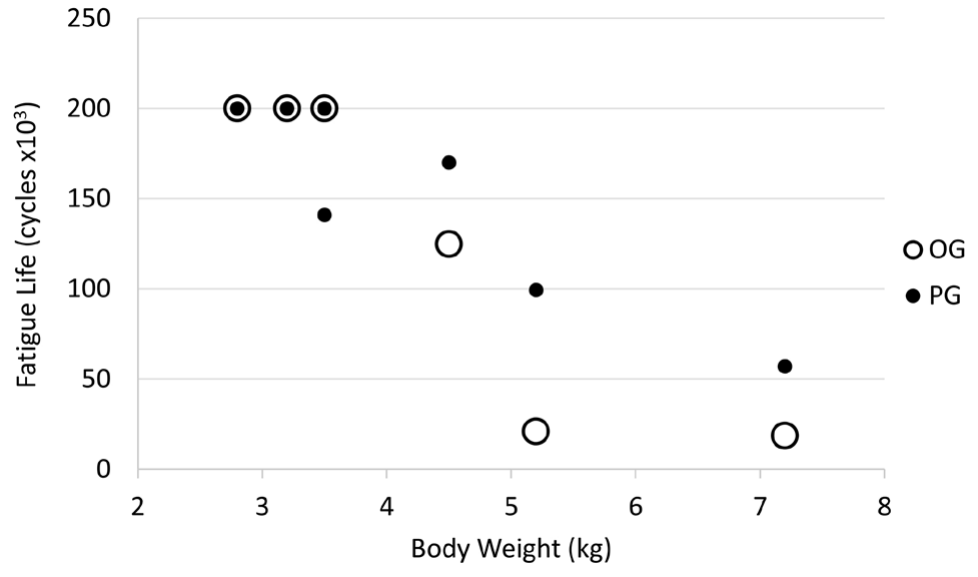
Relationship between dog body weight and fatigue life. Open circle = OG and filled circle = PG.

**Figure 9:**
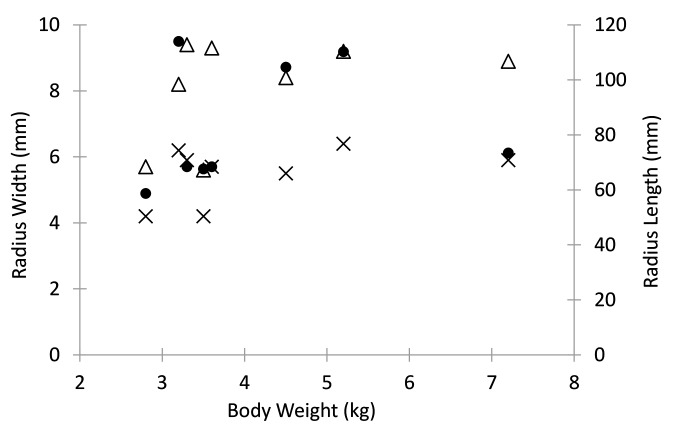
Dog body weight relative to radius cranio-caudal width (X), medio-lateral width (triangle), and length (dot).

There was an inverse relationship between body weight and fatigue life with an apparent decrease in fatigue life with increase in dog weight (R = −0.89; *p* < 0.001; Figure 8). There was no statistical significant relationship between body weight to mediolateral width (R = 0.42, *p* = 0.30), craniocaudal width (R = 0.42, *p* = 0.30) or length (R = 0.18, *p* = 0.67) of the radii Figure 9).

## Discussion

The results of this study indicated that the fatigue properties, but not the residual strength, of distal radial fractures in toy-breed dogs stabilized with a 1.5 mm condylar LCP will be compromised if an open plate hole is placed directly over the fracture gap (OG) versus proximal to the fracture gap (PG). Failure during fatigue and residual strength tests occurred most commonly at the level of the plate. Of paired constructs that had at least one failure before 200,000 cycles, OG constructs failed in fewer cycles than their contralateral PG constructs. OG constructs had lower stiffness and higher gap motion in the first 1,000 cycles than PG constructs.

A small gap model was used in this study whereas a gap is only occasionally present clinically. In locking plates, filling open screw holes with an insert did not increase torsional or axial stiffness of plate constructs (14). A small gap model allowed us to observe that buttressing an open screw hole over bone added protection to the 1.5 condylar LCP compared to an unbuttressed hole over the gap. Results of this study are likely to be different if the model was anatomically reduced and the radius shared the load.

Only 3 screws were used in the proximal bone segment. Studies show that only 3 locking screws per fracture segment are needed to maintain axial stiffness of a locking construct and additional screws are not beneficial (9, 15). Length of the plate and position of the screws near the small gap were chosen to minimize gap strain (9, 15). None of the failures in the current study involved the proximal screws.

Gap motion during cyclic loading was less for PG than OG constructs but both constructs exceeded strains conducive to bone healing. Both constructs had caudal gap strains 1.5–2.5 times the upper limit for indirect bone healing to occur in the first 1,000 cycles. When a fracture gap remains after fracture stabilization, either due to poor reduction techniques or a comminuted fracture, the 1.5 mm condylar LCP will likely not provide sufficient stabilization for fracture healing in toy breed dogs.

PG constructs performed better in fatigue testing compared with OG constructs. Effort to have bone buttressing an open hole near the fracture should be a priority when using the 1.5 mm condylar LCP in distal radius and ulna fractures. Although it is intuitive that stiffness of a construct can be enhanced by avoiding an open screw hole over a fracture line, until this study, there was no evidence that moving that open hole and buttressing it with bone improved this plate’s biomechanical properties in a small gap model.

While residual strength yield and failure loads did not significantly differ between PG and OG constructs, it is pertinent to note that yield loads occurred at loads only marginally higher than approximated trot loads (OG, 66.2 ± 10.6 n; PG: 59.3 ± 11.7 n; Trot Load, 44.4 ± 15.2 n). Controlled activity in the post-operative period would be important to a successful outcome.

Fatigue life of the plate was unacceptable in dogs weighing over 3.5 kg. In dogs over 5 kg, the *in vitro* fatigue life was comparable to only 1.5–2 months of the bone healing period16 with the 1.5 mm condylar LCP stabilization. While surgeons are unlikely to consider use of a 1.5 mm condylar LCP in most 5 kg dogs, it is important to note that radial size does not necessarily correlate to body weight. Our study included similar size radii from dogs with body weights varying between 3–5 kg. Therefore, while radial geometry might be appropriate for a 1.5 mm condylar LCP, body condition score should be considered.

The largest limitation to the current study is the small sample size. Unfortunately, sources for toy breed cadavers were much more limited than expected. Consequently, the power to determine biologically relevant effect sizes was small. Note that mean values for some residual strength variables differed by up to 100%, which could be clinically important, but were without statistical significance in the small sample studied. Confidence intervals for mean residual strength values are reported to enhance interpretation of the sample size limitations. Secondly, the open screw hole proximal to the fracture would likely have been filled with a screw in clinical circumstances. However, filling the hole with a screw would most likely have resulted in even greater stability for the proximal gap group, consistent with the findings in the current study.

Our results indicate that cranial application of a 1.5 mm condylar LCP in which an open cortex hole is positioned proximal to, rather than directly over, the fracture line will improve the performance of the 1.5 mm condylar LCP when used in a non-anatomically reduced distal radius and ulna fracture. The 1.5 mm condylar LCP will not be sufficient stabilization in dogs weighing >3.5 kg even with appropriate radial geometry for the plate.

## Author Contributions

SA: Student in the STAR program. Participated in specimen preparation for mechanical testing, all mechanical test, involved in data reduction and manuscript writing, AK: Co primary mentor. Study idea and participated in design, specimen preparation for mechanical testing, data review, manuscript writing and review and final manuscript preparation for submission. TG: Engineer on the study, mechanical design, specimen preparation and potting, mechanical testing, data manipulation, statistical analysis, manuscript contributions and writing JM: Study design, specimen preparation, review of data and manuscript. SS: Co primary mentor. Study design, mechanical testing, statistical analysis, data review, manuscript writing and review.

## Conflict of Interest Statement

The authors declare that the research was conducted in the absence of any commercial or financial relationships that could be construed as a potential conflict of interest.

## Footnotes

http://synthes.vo.llnwd.net/o16/LLNWMB8/US%20Mobile/Synthes%20North%20America/Product%20Support%20Materials/Technique%20Guides/SUTG1.5ModLCPSysJ12086A.pdf
