# Supplementary material for: Open Screw Placement in a 1.5 mm LCP Over a Fracture Gap Decreases Fatigue Life
Source: Front Vet Sci. 2018 May 23;5:89. doi: 10.3389/fvets.2018.00089 (PMC5975469; doi:10.3389/fvets.2018.00089)
Supplement: Supplementary file 1 [file Table1.docx]

*Supplemental Table 1 – Residual strength test values for individual OG and PG constructs.*

| Variable | Model | Dog | | | |
| --- | --- | --- | --- | --- | --- |
|  |  | 3 | 5 | 6 | 7 |
| Pre-yield stiffness (N/mm) | OG | 30.7 | 8.4 | 44.6 | 58.1 |
|  | PG | 129.5 | 11.6 | NA | 63.0 |
| Yield |  |  |  |  |  |
| Displacement (mm) | OG | 3.1 | 0.7 | 1.6 | 1.8 |
|  | PG | 0.5 | 0.3 | NA | 1.5 |
| Force (N) | OG | 101.0 | 8.7 | 66.1 | 96.0 |
|  | PG | 67.1 | 5.9 | NA | 90.7 |
| Energy (kN*mm) | OG | 160.0 | 4.0 | 67.6 | 102.6 |
|  | PG | 19.8 | 0.8 | NA | 84.7 |
| Maximum |  |  |  |  |  |
| Displacement (mm) | OG | 9.9 | 5.6 | 8.9 | 5.6 |
|  | PG | 5.8 | 3.8 | NA | 6.3 |
| Force (N) | OG | 816.0 | 100.8 | 284.5 | 418.3 |
|  | PG | 443.4 | 113.8 | NA | 374.5 |
| Energy (kN*mm) | OG | 3201.9 | 320.0 | 897.4 | 902.0 |
|  | PG | 1260.3 | 315.0 | NA | 1086.1 |
| Failure |  |  |  |  |  |
| Displacement (mm) | OG | 10.7 | 5.7 | 9.5 | 6.4 |
|  | PG | 6.8 | 3.9 | NA | 7.7 |
| Force (N) | OG | 597.1 | 101.6 | 229.9 | 364.1 |
|  | PG | 684.1 | 77.0 | NA | 327.0 |
| Energy (kN*mm) | OG | 3724.3 | 321.7 | 1058.8 | 1226.7 |
|  | PG | 1884.3 | 326.5 | NA | 1536.6 |
